# Microglia orchestrate synaptic and neuronal stripping: Implication in neuropsychiatric lupus

**DOI:** 10.1111/jcmm.18190

**Published:** 2024-03-17

**Authors:** Yishan Zhou, Liang Chen, Xiulan Zheng, Qijun Fang, Yunzhi Qian, Tianshu Xu, Jun Liang, Huajun Zhang, Xiaojuan Han, Lingyun Sun

**Affiliations:** ^1^ Department of Rheumatology and Immunology, Nanjing Drum Tower Hospital, the Affiliated Hospital of Nanjing University Medical School Nanjing Drum Tower Hospital Clinical College of Nanjing University of Chinese Medicine Nanjing Drum Tower Hospital Clinical College of Nanjing Medical University Nanjing China; ^2^ Department of Gynecology The First Affiliated Hospital of Nanjing Medical University Nanjing China; ^3^ School of Pharmacy Macau University of Science and Technology Macau China; ^4^ Department of Traditional Chinese Medicine, Nanjing Drum Tower Hospital Nanjing Drum Tower Hospital Clinical College of Nanjing University of Chinese Medicine Nanjing China; ^5^ Department of Nutrition, Gillings School of Global Public Health University of North Carolina at Chapel Hill Chapel Hill North Carolina USA

**Keywords:** systemic lupus erythematosus, neuropsychiatric lupus, microglia, M1 phenotype, synaptic stripping, cerebrospinal fluid

## Abstract

Systemic lupus erythematosus (SLE), a multifactorial autoimmune disease, can affect the brain and cause neuropsychiatric dysfunction, also named neuropsychiatric lupus (NPSLE). Microglial activation is observed in NPSLE patients. However, the mechanisms regulating microglia‐mediated neurotoxicity in NPSLE remain elusive. Here, we showed that M1‐like proinflammatory cytokine levels were increased in the cerebrospinal fluid (CSF) of SLE patients, especially those with neuropsychiatric symptoms. We also demonstrated that MRL/lpr lupus mice developed anxiety‐like behaviours and cognitive deficits in the early and active phases of lupus, respectively. An increase in microglial number was associated with upregulation of proinflammatory cytokines in the MRL/lpr mouse brain. RNA sequencing revealed that genes associated with phagocytosis and M1 polarization were upregulated in microglia from lupus mice. Functionally, activated microglia induced synaptic stripping in vivo and promoted neuronal death in vitro. Finally, tofacitinib ameliorated neuropsychiatric disorders in MRL/lpr mice, as evidenced by reductions in microglial number and synaptic/neuronal loss and alleviation of behavioural abnormalities. Thus, our results indicated that classically activated (M1) microglia play a crucial role in NPSLE pathogenesis. Minocycline and tofacitinib were found to alleviate NPSLE by inhibiting micrglial activation, providing a promising therapeutic strategy.

## 1 INTRODUCTION

Systemic lupus erythematosus (SLE) is a chronic autoimmune disease characterized by excessive autoimmune activation, which causes the immune system attacks many tissues, especially the kidney and brain.^1^ Brain involvement occurs in 40%–90% of SLE patients, and the associated neuropsychiatric dysfunction, also known as neuropsychiatric lupus (NPSLE), represents one of the major causes of mortality in SLE patients.^1^, ^2^ Patients with primary and diffuse NPSLE present with a variety of symptoms that range from anxiety, depression, delirium and cognitive impairment to psychosis.^2^ These central nervous system (CNS) symptoms negatively affect patients' quality of life. While the exact aetiology of NPSLE remains largely unclear, certain autoantibodies, immune cell infiltrates and cytokines have been reported to participate in the pathological process of both primary and diffuse NPSLE.^3^ Moreover, the agents currently used to control immune cell activation or autoantibody levels show fewer neuroprotective effects but many side effects.

Although SLE is a multifactorial disease, neuropsychiatric symptoms are typically observed in patients in an early stage of the disease.^4^, ^5^ In particular, in a lupus mouse model, psychiatric dysfunction is detected prior to serological pathology.^6^ These findings suggest that CNS cells/factors rather than peripheral autoimmune cells/mediators are involved in the disease. Correspondingly, strategies that target a specific cell type in the brain may reduce off‐target toxicity and be more efficacious in the treatment of NPSLE than some drugs currently used to treat lupus, which induce broad immunosuppression and cytotoxicity. Thus, there is an urgent need to identify key mediators in the brain and develop targeted therapeutic strategies for NPSLE.

Microglia, resident macrophages within the brain, are the predominant immune cells in the CNS. Microglia‐mediated neuroinflammation plays an important role in the pathogenesis of many neurodegenerative diseases.^7^ Increased microglial activation has been reported in the brains of NPSLE patients and lupus mice.^8^, ^9^, ^10^ Depending on the microenvironment, microglia can adopt one of two major phenotypes, termed M1 (classical activation) and M2 (alternative activation).^11^, ^12^ Functionally, M1 microglia produce many proinflammatory factors and release them into the extracellular milieu, eventually causing damage to surrounding neurons.^13^, ^14^ In addition to playing an inflammatory role, classically activated microglia exhibit increased phagocytosis, through which they engulf dendritic spines and contribute to synapse loss in mild/early forms of many mental disorders.^15^, ^16^ These findings indicate that targeting microglial activation has therapeutic potential for neuroinflammatory‐related diseases.

During the development of SLE and M1 microglial polarization, the Janus kinase (JAK) family plays important roles in cytokine signalling and phenotype induction,^17^, ^18^ which indicates that small‐molecule inhibitors of JAKs may have therapeutic potential for NPSLE. Tofacitinib, a Food and Drug Administration (FDA)‐approved nonselective JAK inhibitor, has been used to treat rheumatoid arthritis,^19^ ulcerative colitis^20^ and psoriatic arthritis^21^ in many experimental studies and clinical trials. Our previous study showed that tofacitinib could ameliorate lupus nephritis (LN) in lupus mice by increasing the expression of TGFbRI and suppressing the activation of CD4^+^ T cells.^22^ However, the contribution of tofacitinib to microglial polarization and neuropsychiatric dysfunction in SLE remains unknown.

In the present study, we utilized CSF samples from SLE patients and mice of the MRL/lpr strain, a validated lupus‐prone model, to investigate the mechanism of microglial activation in NPSLE. We revealed that the levels of M1‐associated proinflammatory cytokines were increased in the CSF of NPSLE patients and that classically activated microglia were involved in synaptic/neuronal stripping in the brains of lupus model mice. Importantly, we found that minocycline and tofacitinib inhibited microglial activation‐mediated synaptic engulfment and inflammatory neurotoxicity in vivo and in vitro. Moreover, both minocycline and tofacitinib significantly ameliorated neuropsychiatric dysfunction in MRL/lpr mice. Collectively, our results provide a promising therapeutic strategy for NPSLE.

## 2 MATERIALS AND METHODS

### 2.1 Study approval and human subjects

In total, 14 patients with NPSLE, 9 SLE patients without NP manifestations (non‐NPSLE patients) and 6 controls (healthy volunteers or encephalitis patients) from the Department of Rheumatology and Immunology of the Affiliated Drum Tower Hospital of Nanjing University Medical School were enrolled as we previously reported.^23^ Clinical diagnosis was assessed according to the American College of Rheumatology (ACR) revised SLE criteria^24^ and nomenclature and case definitions for NPSLE.^25^ Specially, patients with diffuse neuropsychological manifestations (dNPSLE, clinical symptoms including mood or memory disorder, psychosis and delirium) were included. The study was approved by the Ethics Committee of our institute (ID: SC201700201), and written informed consent for the collection of cerebrospinal fluid (CSF) was obtained from all participants.

### 2.2 Mice and treatments

Female MRL/MpJ‐Fas^lpr^ (MRL/lpr) and MRL/MpJ (MRL/mpj) mice were obtained from Shanghai Lingchang Biotechnology Corporation (Shanghai, China). Newborn or pregnant C57BL/6 mice were obtained from the Model Animal Research Center of Nanjing University (Nanjing, China). All animals were maintained under specific pathogenfree and standard laboratory conditions. To inhibit phagocyte activation, the MRL/lpr mice were randomly divided and treated with minocycline (50 mg/kg/d, Sigma‐Aldrich, M2280000; daily by i.p. injection for 3 weeks starting at the fifth week)^26^ or PBS. For JAK inhibition, the MRL/lpr mice were treated with tofacitinib (15 mg/kg/d, Abmole Bioscience, CP‐690550; daily by oral gavage for 10 weeks starting at the 10th week)^22^ and vehicle (0.5% methylcellulose/0.025% Tween 20 in ddH_2_O). All experiments were approved by the Ethics Committee for Animal Research of the Affiliated Drum Tower Hospital of Nanjing University Medical School (No. 2019AE01084).

### 2.3 Olink cytokine measurements

The relative concentrations of inflammatory cytokines in CSF from SLE or control individuals were analysed by multiplex proximity extension (MPE) assay with the provided Inflammation Probe Panel assay of 92 analytes (Olink Bioscience, Uppsala, Sweden) detected by a Fluidigm Biomark reader (Fluidigm Corporation, USA) following the vendor's (LC‐Bio Technology CO., Ltd., Hangzhou, China) recommended protocol. The data are reported as normalized protein expression values (NPX, Olink Proteomics arbitrary unit on a log2 scale) as described.^27^ Significance was assessed using the *t*‐test (*p* < 0.05).

### 2.4 Tissue imaging and immunofluorescence analysis

The brain tissues were embedded in OTC and sectioned at 25 μm using a freezing microtome. For fluorescence immunostaining, brain sections were rinsed with PBS, permeabilized with PBS containing 0.3% Triton X‐100 (PBST), blocked with blocking buffer (5% bovine serum albumin in PBST) for 1 h and incubated with primary antibodies (IBA‐1, 1:500, Wako, 019–19741; PSD‐95, 1:500, Abcam, ab2723; NeuN, 1:500, Abcam; ab177487) overnight. Sections were then washed, incubated with secondary antibodies and mounted using ProLong Diamond medium (Invitrogen, PK401). For neuron and microglia quantification, stained samples were scanned using a Olympus FV3000 confocal laser microscope and quantitatively analysed by the optical fractionator method (Stereo Investigator software, Microbrightfield) as described.^28^ For microglial engulfment analysis, sections stained for IBA‐1 and PSD‐95 were imaged using an Olympus SpinSR spinning disk confocal microscope. IBA‐1^+^ microglia were then 3D reconstructed with Imaris software (Bitplane, Zurich, Switzerland), and synaptic puncta inside the IBA‐1^+^ phagocytes were quantified using the spot rendering function as described.^29^

### 2.5 Golgi staining

For dendritic spine quantification, brain sections were stained using the FD Rapid Golgi Stain kit (FD Neuro Technologies, PK401) according to the manufacturer's instructions. Golgi‐stained neurons and dendritic segments from the hippocampus were imaged under a microscope (FV3000 Microscope, Olympus) with a 100× objective. Dendritic branching and spines were analysed using NIH ImageJ software as we previously described.^29^

### 2.6 Ex vivo isolation of microglia

Ex vivo microglia isolation was performed as described.^30^ Deeply anaesthetised mice were intracardially perfused with ice‐cold PBS. Brain tissues were minced and enzymatically digested in DMEM/F12 containing 2% FBS, 0.5 mg/mL collagenase type IV (Sigma‐Aldrich) and 20 U/mL DNase I (Sigma‐Aldrich) at 37°C for 1 h with shaking. The homogenates were then filtered with a strainer (70 μm) and centrifuged at 500 g for 10 min. Cell pellets were resuspended in 4 mL of a 37% Percoll solution (GE Healthcare, 17‐0891‐09). The pellets containing microglia were obtained by density gradient centrifugation and resuspended in MACS buffer. CD11b^+^ Microglia were isolated using manual MACS sorting (Miltenyi Biotec, 130‐093‐636) according to the manufacturer's instructions.

### 2.7 RNA sequencing

RNA sequencing of isolated microglia was conducted as reported.^29^ Total RNA was extracted from microglia using Trizol reagent (Thermo Fisher) and mRNA was purified and reverse transcribed to create the cDNA templates, which were next used to synthesize the final cDNA libraries. 2 × 150‐bp paired‐end sequencing (PE150) was performed using the Illumina Novaseq™ 6000 sequence platform (LC‐Bio Technology Co., Ltd., Hangzhou, China) according to the vendor's recommended protocol. Reads obtained from the sequencing analyses were filtered by Cutadapt and aligned to the murine reference genome using HISAT2 package. DEGs (|log2FC|≥1 and *q* < 0.05) were analysed using DESeq2 software. DEGs were then subjected to enrichment analyses of GO functions and KEGG pathways.

### 2.8 Cell culture and treatment

Primary microglia and neurons were obtained from postnatal (P1 to P2) and embryonic (E14/15) mice, respectively, as described.^31^ In brief, cortex tissues were dissected, mechanically dissociated, digested with trypsin and filtered through a 70 μm filter to obtain a single cell suspension. For microlgia culture, the obtained cells were plated on poly‐L‐lysine‐precoated flasks and cultured in DMEM supplemented with FBS (10% v:v) and penicillin/streptomycin (1% v:v). 1–2 weeks later, microglia were dissociated by shaking the flasks and plated on poly‐L‐lysine‐precoated 24‐well plates. For M1 phenotype induction, microglia were treated with 100 ng/mL LPS (Sigma‐Aldrich) and 20 ng/mL IFNγ (Peprotech) for 24 h. For pharmacological measurements, the JAK inhibitor tofacitinib (TOF, 10 μM) was added 1 h before stimulation. For primary neuron culture, filtered and obtained single cells were plated on poly‐L‐lysine‐precoated 96‐/24‐well plates and cultured with Neurobasal medium (21103049, Gibco) supplemented with B27 (2% v:v, Gibco, 17504044) and penicillin/streptomycin (0.5% v:v). For toxicity assessments, primary neurons were treated with microglia‐conditioned media (MCM) mixed with the neurobasal culture medium (1:2) for 12 h.

### 2.9 Cell viability CCK‐8 assay

The changed cell viability of MCM treatment was detected by cell counting kit‐8 (CCK‐8 Kit, c0037, Beyotime, Shanghai, China) as reported.^31^ In brief, cortical neurons were seeded in 96‐well plate and then treated with MCM for 12 h. Then 10 μL of CCK‐8 reagent was added to each well for 4 h. Finally, the absorbance was detected by the Multiskan Spectrum (Thermo Fisher Scientific) at 450 nm.

### 2.10 Quantitative real‐time PCR

Real‐time PCR was performed using StepOnePlus Real‐Time PCR Systems (Applied Biosystem). Total RNA was extracted from cortex tissues using TRIzol reagent (Vazyme Biotech, R401‐01). cDNA was synthesized using a HiScript III RT SuperMix for qPCR Kit (Vazyme, R323‐01). Next, the cDNAs were amplified and quantified using ChamQ SYBR qPCR Master Mix (Vazyme, Q341‐02). *Gapdh* was used as an internal control. Primer sequences used for qPCR were as follows:

mGapdh F: CAAAAGGGTCATCTCC; R: CCCCAGCATCAAAGGTG.

mIl6 F: CCCCAATTTCCAATGCTCTCCT; R: CATAACGCACTAGGTTTGCCG. mTnfα F: CCCACGTCGTAGCAAACCA; R: GGCAGAGAGGAGGTTGACTT. mCcl3 F: ATGCAAGTTCAGCTGCCTGC; R: ATGCCGTGGATGAACTGAGG.

### 2.11 Mouse behavioural testing

Behavioural tests was monitored through a video camera and analysed with a video tracking system (Top Scan software; Top Scan Software & Instruments, USA) as reported.^29^ The elevated‐plus maze (EPM) test and novelty Y maze (NYM) test were performed to assess anxiety‐like behaviour and memory, respectively. EPM consists of two open and two closed arms that extend out from a central platform. Individual mouse was placed in the center platform of the maze and allowed to explore the apparatus for 6 min. The entries in open and closed arm as well as the total time spent in open, closed and center compartments were recorded. Novelty Y maze consisted of a forced choice trial and a free‐choice trial. For forced choice trial, the start arm and one test arm were open, with access to the second test arm blocked. Individual mouse was placed in the start arm and allowed to explore the open test arm for 3 min. For free‐choice trial, the mice were placed back into the Y maze and allowed to explore both the open and test arms for 3 min. For each mouse, the entries in previously non‐accessible arm (the novel arm) and percentage of time exploring the novel arm during the free‐choice trial were calculated.

### 2.12 Statistical analysis

For all statistical analyses, GraphPad Prism 6.0 software was used. Error bars represent SEM in all figures. Analyses used in this study include one‐way ANOVA, or Student's *t*‐test. For ANOVA analyses, Tukey's post hoc tests were subsequently performed. Significance is reported at *p < 0.05, **p < 0.01 and ***p < 0.001.

## 3 RESULTS

### 3.1 The levels of M1‐associated proinflammatory factors were increased in the CSF of NPSLE patients

**FIGURE 1:**
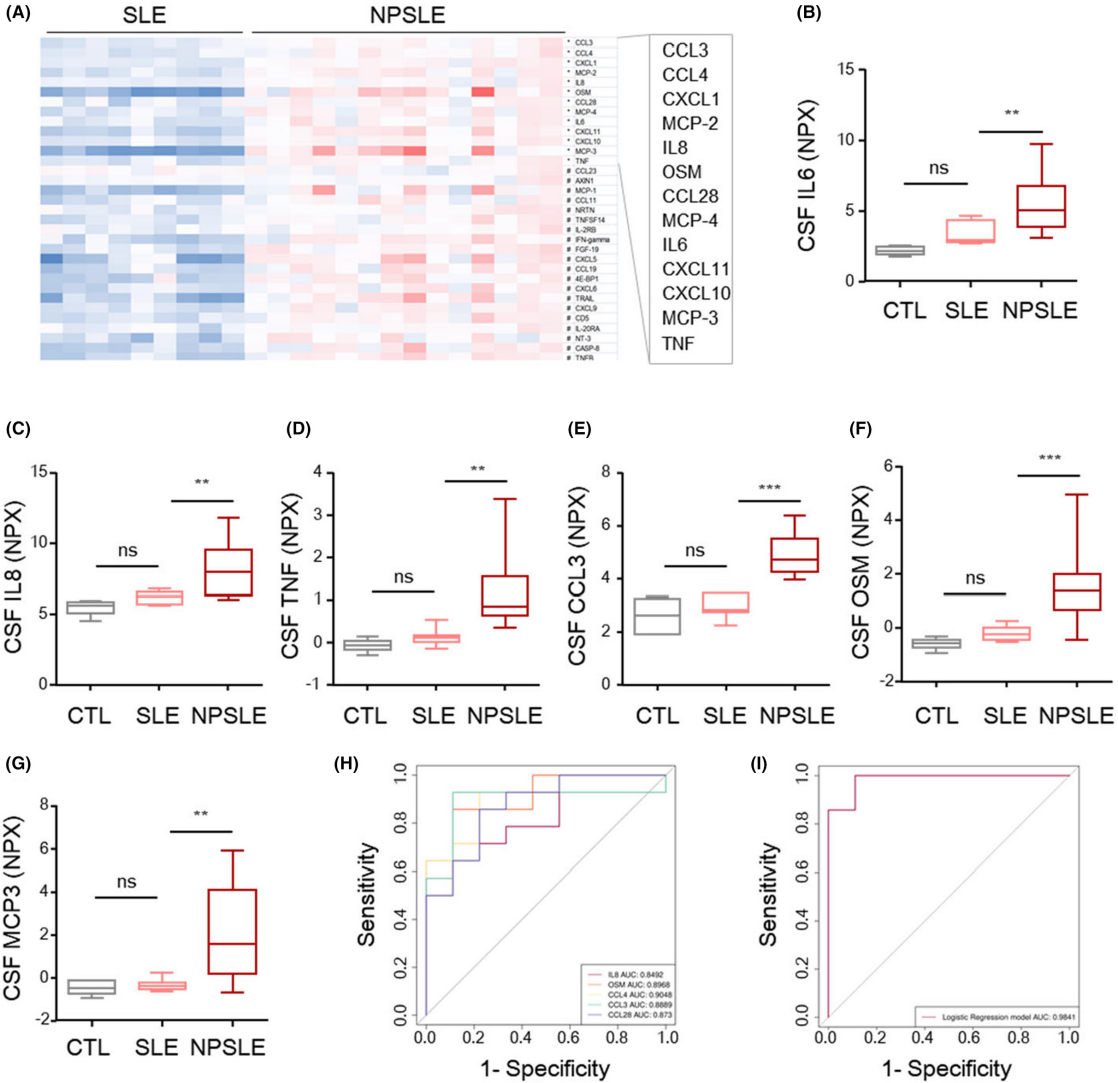
The levels of M1‐associated proinflammatory factors are elevated in the CSF of patients with NPSLE. (A) Screening of significantly upregulated cytokines in the CSF of patients with NPSLE (NPSLE; *n* = 14) and non‐NPSLE patients (SLE; *n* = 9). (B–G) Relative protein levels of IL6, IL8, TNF CCL3, OSM and MCP3 in the CSF of NPSLE patients (*n* = 14), SLE patients (SLE, *n* = 9) and controls (CTL, *n* = 6). (H) Area under the ROC curve (AUC) of the individual top five upregulated cytokines (with the smallest *p*‐value) in the CSF of patients with NPSLE (*n* = 14) compared with that of patients with SLE (*n* = 9). (I) Total ROC curve of the top five cytokines in (H) through logistic regression model. The data are presented as the mean ± SEM. * in (A) adjusted *p* < 0.05; # in (A) *p* < 0.05. **p < 0.01; ***p < 0.001. *p‐*values were determined by *t*‐test (A) or one‐way ANOVA followed by Tukey's post hoc test (B–G). NPX, normalized protein expression values. See also Tables S1 and S2; ns, not significant.

To identify the differentially expressed cytokines in the SLE brain, CSF samples from SLE and control subjects (Table S1) were hybridized with an inflammation panel of 92 predefined cytokines, chemokines and other proteins (Table S2) for a proximity extension assay (PEA). According to the data, 41 of 54 specific proteins (detected in >70% of samples) were differentially expressed between SLE patients and control individuals (adjusted *p* < 0.05, Table S2). Then, to identify the potential factors involved in the pathogenesis of lupus‐related brain injury, we further divided the SLE patients into patients with neuropsychiatric symptoms (NPSLE patients) and patients without neuropsychiatric symptoms (SLE patients, non‐NPSLE patients). Single‐protein logistic regression models revealed that 13 of 54 specific proteins, including OSM, IL8, MCP‐2, CCL4, TNF and other inflammatory factors, were differentially expressed (adjusted *p* < 0.05) between NPSLE and non‐NPSLE patients (Figure 1A). We confirmed that the levels of M1 markers such as IL6, IL8, TNF, CCL3, OSM and MCP3 were significantly increased in the CSF of NPSLE patients compared to that of non‐NPSLE patients (Figure 1B–G). Among the markers, IL8, OSM, CCL4, CCL3 and CCL28 were most strongly associated with a diagnosis of NPSLE, evidenced by the individual ROC curve (Figure 1H) and the total ROC curve through logistic regression model (Figure 1I). These data indicate that the presence of M1‐associated proinflammatory factors in the CSF is associated with neuropsychiatric dysfunction in SLE.

### 3.2 MRL/lpr mice developed anxiety‐like behaviour in the early stage of lupus and cognitive impairment in the active stage of lupus

**FIGURE 2:**
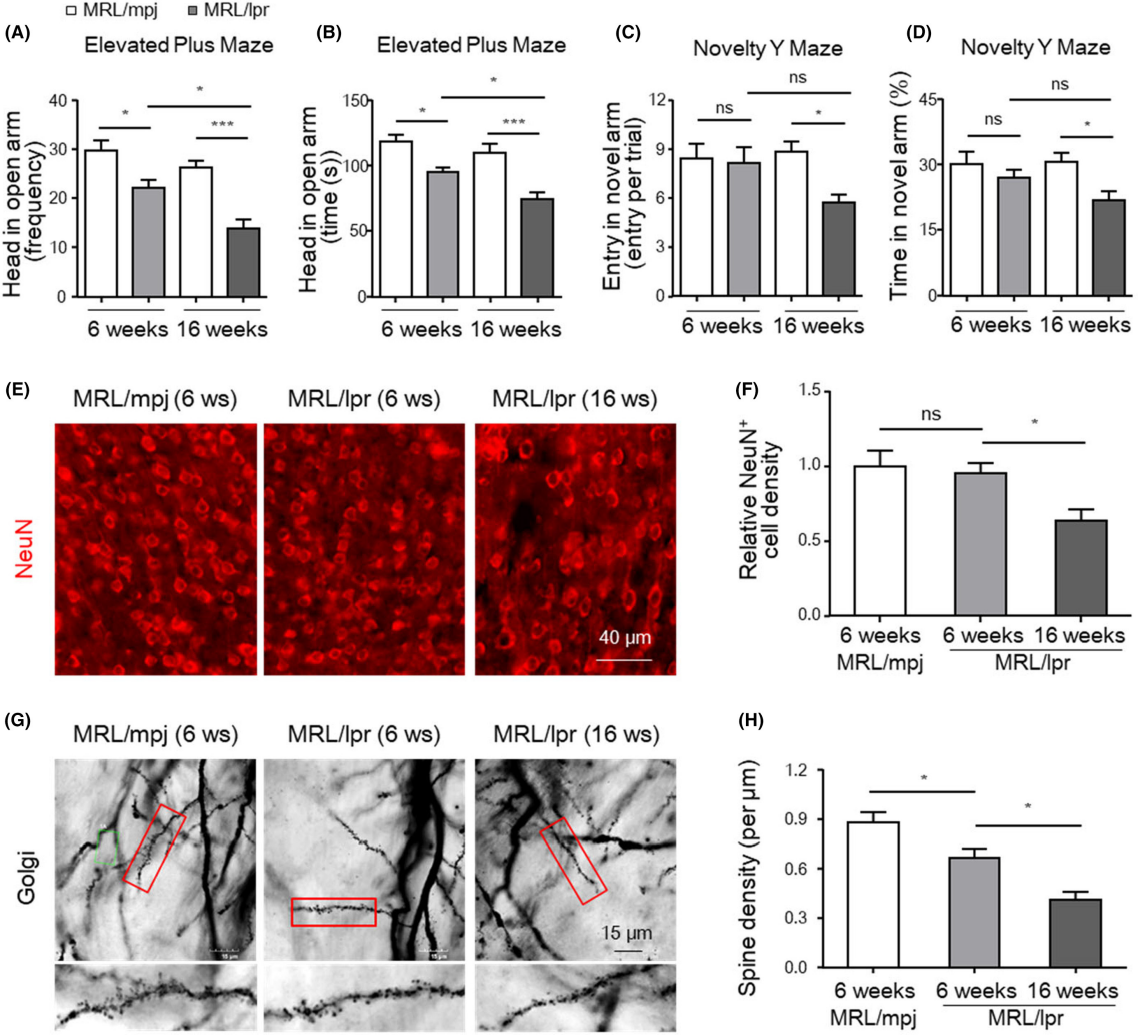
MRL/lpr mice developed anxiety‐like behaviour and cognitive impairment in the early and active stages of lupus. (A–D) Behavioural performance of 6‐week‐old and 16‐week‐old female MRL/lpr mice and matched controls (MRL/mpj) in the EPM and NYM tests (*n* = 8). (E, F) Representative image and quantification of NeuN expression in prefrontal cortex in MRL/mpj and MRL/lpr mice at the indicated time points (*n* = 5). Scale bar, 40 μm. (G, H) Images and quantification of Golgi‐stained dendritic spines in DG granule neurons in MRL/mpj and MRL/lpr mice (*n* = 5). Scale bar, 15 μm. The data are presented as the mean ± SEM. *p < 0.05; ***p < 0.001. *p‐*values were determined by one‐way ANOVA followed by Tukey's post hoc test (A–D, F, H). EPM, elevated‐plus maze; NYM, novelty Y maze; ns, not significant.

In most SLE patients, psychiatric disorders and cognitive impairment occur at different stages of the disease.^3^, ^29^ To assess CNS injury and investigate the underlying mechanisms, we monitored neuropsychiatric changes in the MRL/lpr strain, a well‐established lupus‐prone murine model.^32^, ^33^ MRL/lpr mice developed typical systemic lesions, as evidenced by increased proteinuria and the presence of anti‐dsDNA antibodies in the serum, consistent with our previous reports.^29^, ^34^ We found that MRL/lpr mice exhibited increased anxiety‐like behaviour at 6 weeks of age (the early stage of lupus), as evidenced by the fact that they made fewer number of entries into (Figure 2A) and spent less time in (Figure 2B) the open arms of the elevated‐plus maze (EPM) than wild‐type control animals. Anxiety‐like behaviour was further aggravated at 16 weeks of age (the active stage of lupus, Figure 2A,B). We then assessed cognitive function by the novelty Y maze (NYM) test. We found that MRL/lpr mice behaved similarly to wild‐type control mice at 6 weeks of age but exhibited cognitive impairment at 16 weeks of age (Figure 2C,D).

The prefrontal cortex and hippocampus are the major brain regions responsible for emotion and learning. Stereological counts of neurons, marked by NeuN staining, revealed that there was no difference in the number of neurons in prefrontal cortex in and CA1 region between MRL/lpr and control mice at 6 weeks of age (Figure 2E,F). However, the number of NeuN^+^ neurons in the prefrontal cortex was significantly reduced in MRL/lpr mice at 16 weeks of age (Figure 2E,F). Synaptic defects rather than neuronal death might be involved in mild neuropathy.^35^ Golgi staining revealed that the number of dendritic spines in DG granules was reduced in MRL/lpr mice at 6 weeks of age and that this decrease was further aggravated at 16 weeks of age (Figure 2G,H). These results suggest that synaptic defects and neuronal loss may be causes of early anxiety and later cognitive impairment, respectively, in SLE.

### 3.3 MRL/lpr mice showed increased microglial overactivation and elevated proinflammatory factor levels in the brain

**FIGURE 3:**
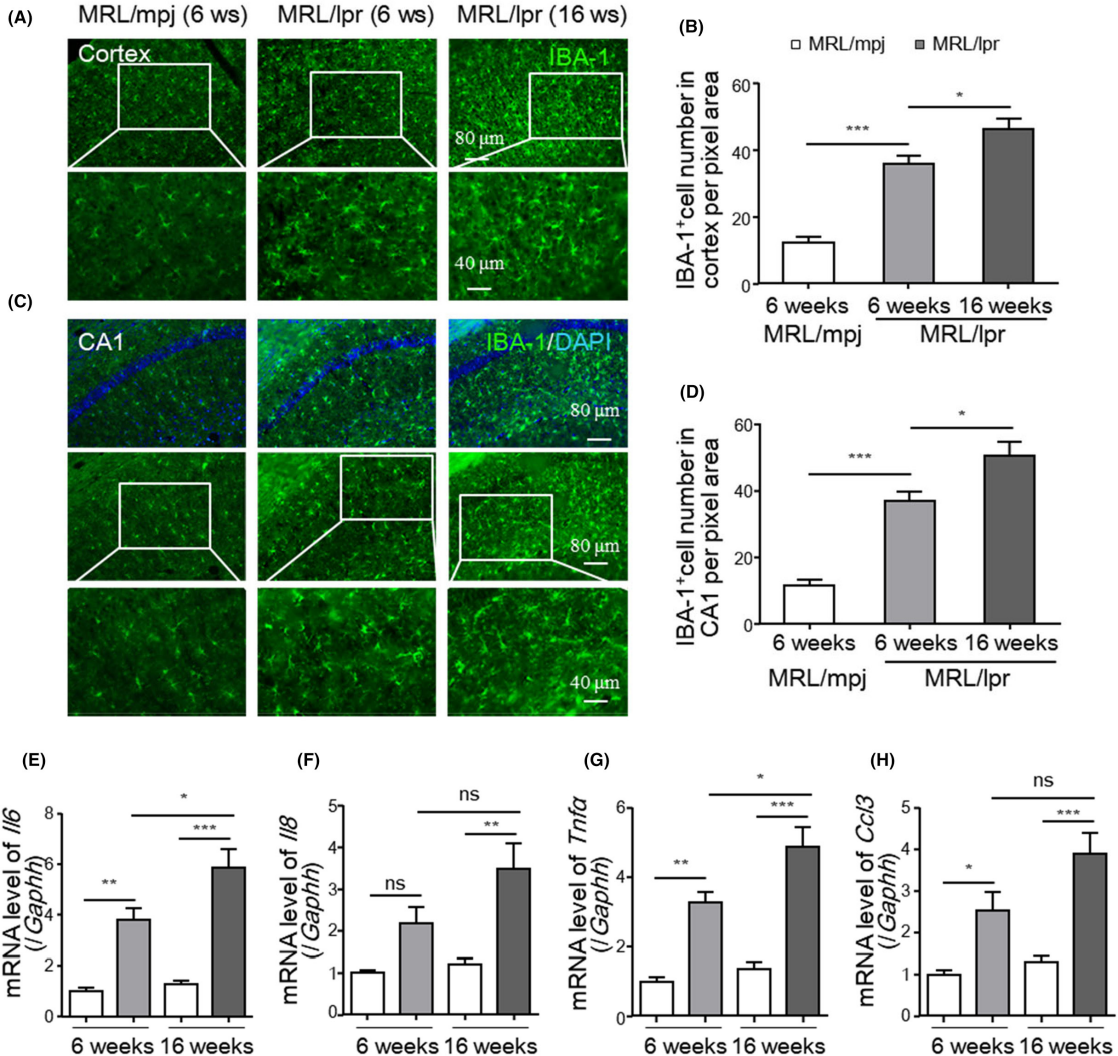
MRL/lpr mice showed increased microglial activation and elevated proinflammatory cytokine levels in the brain. (A–D) Representative IBA‐1 immunofluorescence images (A, C) and the average density of microglia in prefrontal cortex and hippocampal CA1 sections (B, D). Scale bars, 80 and 40 μm under magnification (*n* = 5 mice/group). (E‐H) mRNA levels of *Il6* (E), *Il8* (F), *Tnfα* (G) and *Ccl3* (H) in the cortices of MRL/lpr mice and matched littermate controls (MRL/mpj) (*n* = 5 mice/group). The data are presented as the mean ± SEM. *p < 0.05; **p < 0.01; ***p < 0.001. *p‐*values were determined by one‐way ANOVA followed by Tukey's post hoc test; ns, not significant.

Increased neuroinflammation plays crucial roles in neuronal damage, and microglia are the major mediators of neuroinflammation. We performed immunofluorescence staining of IBA‐1, a microglial marker, to investigate microglial activation. MRL/lpr mice showed extensive microglial overactivation, as evidenced by increases in the number of overactivated microglia with ameboid morphology in the prefrontal cortex and CA1 region (Figure 3A–D). Classical activation (also termed M1 polarization) of microglia is associated with the generation of multiple proinflammatory cytokines.^11^, ^14^ We assessed the mRNA levels of these proinflammatory cytokines in the brains of lupus mice and found that the levels of M1‐associated factors, such as *Il6*, *Il8*, *Tnfα* and *Ccl3*, in the cortex were increased in MRL/lpr mice compared to control mice at 8 weeks of age, although the difference in *Il8* levels was not significant (Figure 3E–H). Moreover, the levels of most of these cytokines increased as the disease progressed (Figure 3E–H). These results were consistent with the changes observed in the CSF of NPSLE patients.

Collectively, these data suggest that lupus mice exhibit microglial overactivation and increased neuroinflammation, which promote neuronal degeneration.

### 3.4 Transcriptional profiling reveals molecular changes in microglia in lupus mice

**FIGURE 4:**
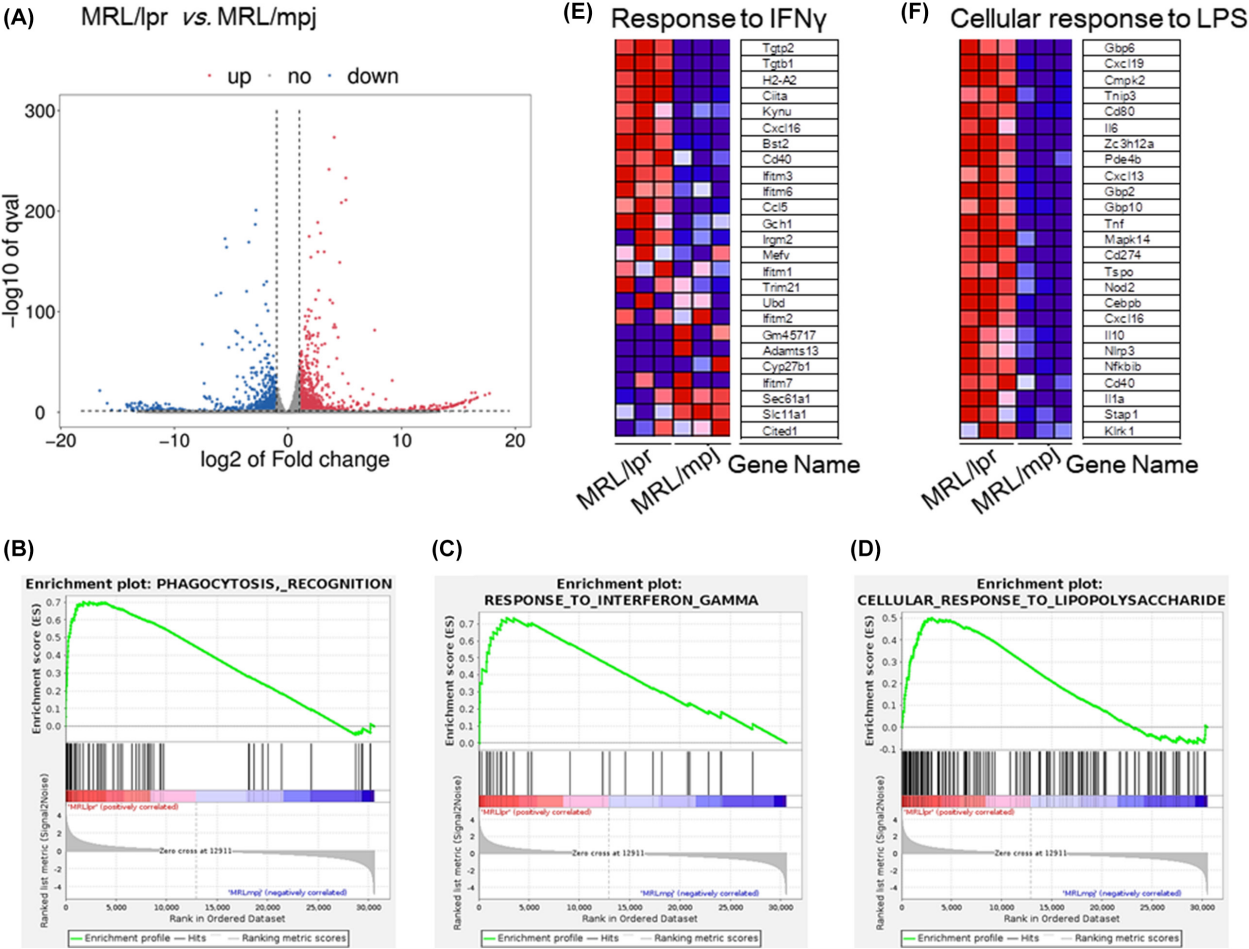
Transcriptional profiling reveals molecular changes in microglia in lupus mice. (A) Volcano plot of genes showing significant changed expression in microglia from MRL/lpr mice compared to those from MRL/mpj mice quantified using RNA‐seq (|log2FC|≥1, *q* < 0.05). (B–D) Results of GSEA showing that phagocytosis, response to IFNγ and cellular response to LPS signalling pathway gene sets were significantly enriched in microglia from MRL/lpr mice compared to those from MRL/mpj (|NES|>1, *p* < 0.05). (E, F) Screening of the top 25 upregulated genes associated with IFNγ (E) and LPS (F) in microglia from MRL/lpr mice (*n* = 3) and control MRL/mpj mice (*n* = 3).

To identify the regulators of microglial overactivation, we sorted microglia from the brains of MRL/lpr and MRL/mpj mice and performed RNA sequencing. Transcriptomics revealed that 1841 transcripts were differentially expressed (890 upregulated, 951 downregulated in MRL/lpr mice, |log2FC|≥1, *q* < 0.05, Figure 4A). GO enrichment analysis revealed that the upregulated RNAs in the microglia of MRL/lpr mice were enriched in immune‐activated pathways (Table S3). We also found that the differentially expressed transcripts were enriched in the pathway of microglial‐mediated phagocytosis (Figure 4B). Lipopolysaccharide (LPS) plus interferon gamma (IFNγ) challenge is commonly used to induce M1 polarization.^36^, ^37^ Indeed, microglia sorted from MRL/lpr mice showed activation of specific pathways in response to IFNγ challenge (Figure 4C) and cellular responses to LPS challenge (Figure 4D). The top 25 upregulated genes associated with IFNγ and LPS in microglia from MRL/lpr mice according to the sequencing data are listed in Figure 4E,F. These data indicate that the microglia of lupus mice showed increased phagocytic activity and characteristics of M1 polarization.

### 3.5 Microglia induced neurodegeneraton in lupus mice via synaptic phagocytosis and neuroinflammatory toxicity

**FIGURE 5:**
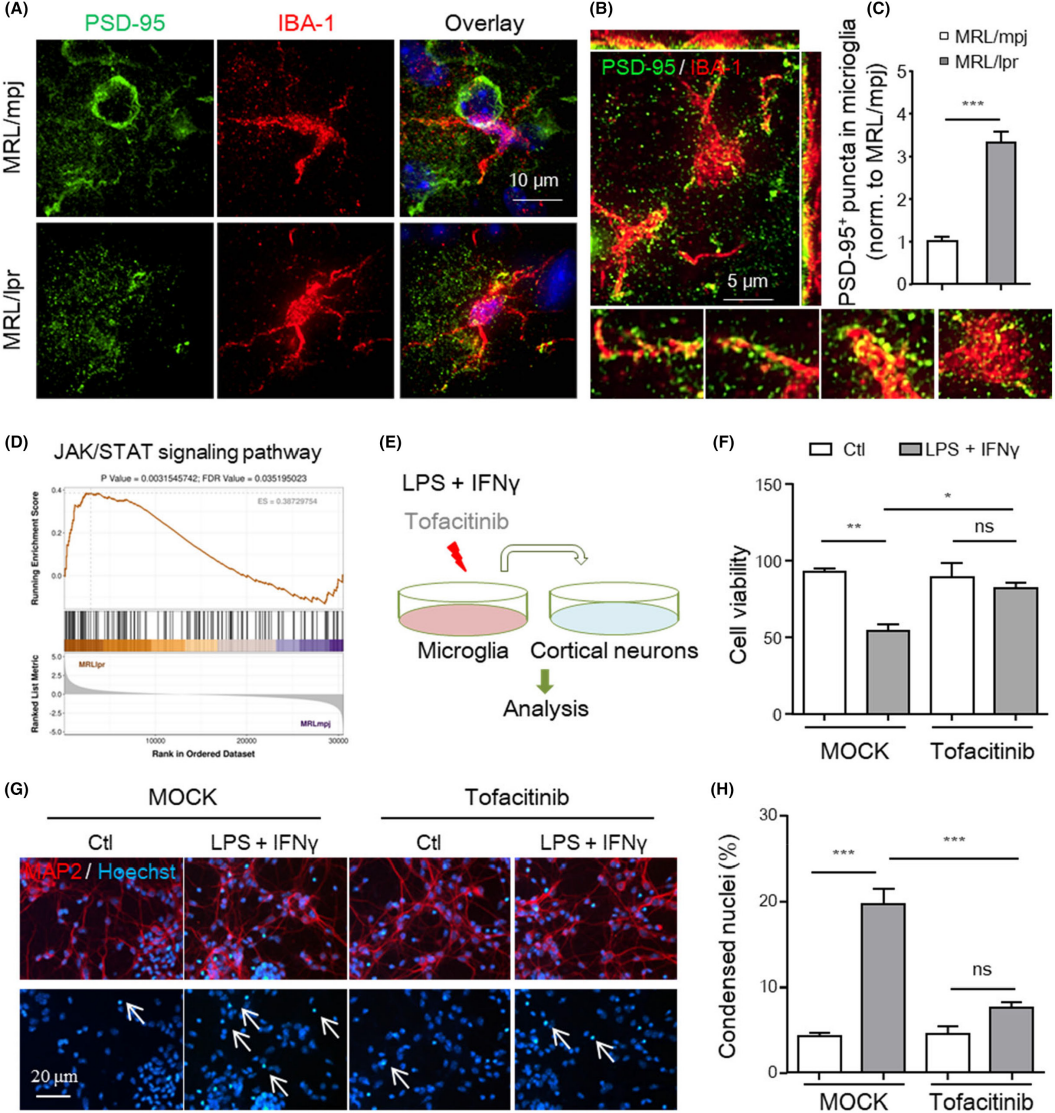
Microglia induced neurodegeneration in lupus via synaptic stripping and neuroinflammatory toxicity. (A–C) Representative immunofluorescence images of postsynaptic puncta (PSD‐95, green) engulfed in IBA‐1^+^ microglia (red) in the pyramidal layer of the CA1 region in brain sections from the indicated mice. Scale bar, 10 μm (A) and 5 μm (B). (C) Quantification of reconstructed PSD‐95^+^ spheres inside microglial cells in the indicated mice (*n* = 18–20 cells from 4 to 5 mice). (D) Results of GSEA showing that JAK/STAT signalling pathway gene sets were significantly enriched in microglia from MRL/lpr mice compared to those from MRL/mpj (|NES|>1, *p* < 0.05). (E‐H) Cultured neurons treated with cultured medium from LPS + IFNγ‐treated microglia for 24 h. (E) Treatment protocol. Primary cortical neurons were incubated with MCM mixed with neurobasal medium for 12 h, and then analysis was performed. (F) The viability of primary neurons was assayed by the CCK8 assay. (G, H) Images of primary neurons stained with Hoechst and quantitative analysis of the Hoechst positive cells with condensed nuclei. Scale bar, 20 μm. The data are shown as the mean ± SEM of three independent experiments. *p < 0.05; **p < 0.01; ***p < 0.001; one‐way ANOVA followed by Tukey's post hoc test. LPS, lipopolysaccharide; MCM, microglia‐derived cultured medium; ns, not significant; TOF, tofacitinib.

To confirm the effects of microglial overactivation on neuronal damage observed in the brains of lupus mice, synaptic phagocytosis was evaluated in vivo and microglia‐mediated neurotoxicity was investigated in vitro. Activated microglia can engulf or prune synapses.^38^, ^39^ MRL/lpr mice showed synaptic defects in the hippocampus at 6 weeks of age (Figure 2G,H). To directly measure synaptic engulfment by microglia, we performed high‐resolution imaging and 3D reconstruction of hippocampal sections from MRL/lpr mice. As shown in Figure 5A–C, both imaging and quantitative analysis revealed that the number of PSD‐95^+^ puncta within IBA‐1^+^ microglia was significantly higher in MRL/lpr mice than in control mice. These results indicate that increased microglial phagocytosis accounts for synaptic loss in MRL/lpr mice.

Microglia‐derived proinflammatory factors exert neurotoxic effects in many disorders.^11^, ^12^ We next investigated whether microglial polarization accounts for the neuronal loss observed in the cortices of MRL/lpr mice. To this end, primary cortical neurons (MAP^+^) were cultured, and LPS plus IFNγ‐challenged microglia‐conditioned medium (MCM) was added (Figure 5E). As shown, the addition of LPS + IFNγ‐stimulated MCM led to a significant reduction in the viability of cultured cortical neurons (Figure 5F).

JAK acts downstream of both LPS and IFNγ.^18^, ^40^ Indeed, we found that microglia sorted from MRL/lpr mice showed activation of JAK/STAT signalling pathway (Table S4 and Figure 5D). Moreover, as shown in Figure 5F–H, the neurotoxic effects of LPS + IFNγ‐treated microglia were significantly blunted by tofacitinib, a nonselective JAK inhibitor, suggesting that JAK blockade can reverse the toxic effects of M1 microglia on neurons. The data suggest that excessive microglial overactivation accounts for both synaptic loss and neuronal death in the brains of lupus mice.

### 3.6 Minocycline and tofacitinib ameliorated neuropsychiatric symptoms and neurodegeneration in MRL/lpr mice

**FIGURE 6:**
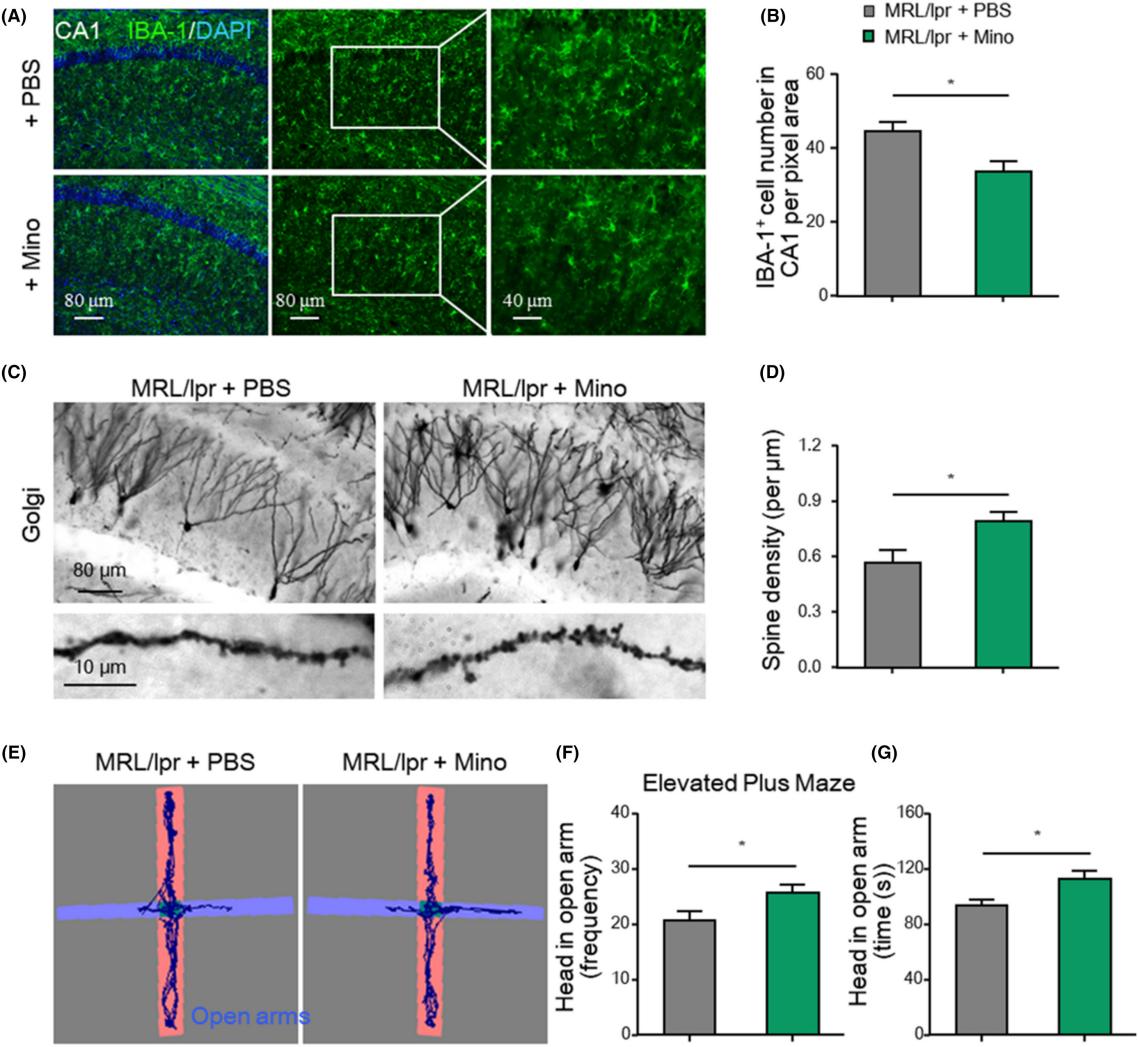
Minocycline ameliorated the neuropsychiatric symptoms and neurodegeneration in MRL/lpr mice. (A, B) Representative images and quantification of IBA‐1^+^ microglia in the hippocampi of the indicated mice (*n* = 5). Scale bars as indicated. (C, D) Representative images and quantification of synapses by Golgi staining in hippocampal sections (*n* = 5). Scale bars as indicated. (E–G) Frequency of entries and time spent with the head in the open arms in the EPM test (*n* = 7). Total time in the maze: 6 min. The data are presented as the mean ± SEM. *p < 0.05; by *t*‐test (B, D, F, G). EPM, elevated‐plus maze; mino, minocycline.

Our findings indicate that targeting microglial overactivation may be a potential strategy for treating neuropsychiatric disorders in lupus. Minocycline is a BBB‐permeable drug that can suppress microglial activation and phagocytic activity.^41^ To determine whether minocycline exerts neuroprotective effects in lupus, MRL/lpr mice were treated with minocycline beginning at 5 weeks of age and then behavioural tests were performed 3 weeks later. As microglial phagocytosis‐mediated synaptic loss is essential for the development of early anxiety‐like behaviours, we investigated related changes in minocycline‐treated mice. As shown in Figure 6A–D, minocycline treatment significantly suppressed microglial activation, as evidenced by reduced cell density (Figure 6A,B) and alleviated synaptic loss (Figure 6C,D). Then, we evaluated the anti‐anxiety effect of minocycline by the EPM test. The number of entries into (Figure 6E,F) and time spent in (Figure 6E,G) the open arms of the EPM were significantly increased in the minocycline‐treated group compared with the control group.

**FIGURE 7:**
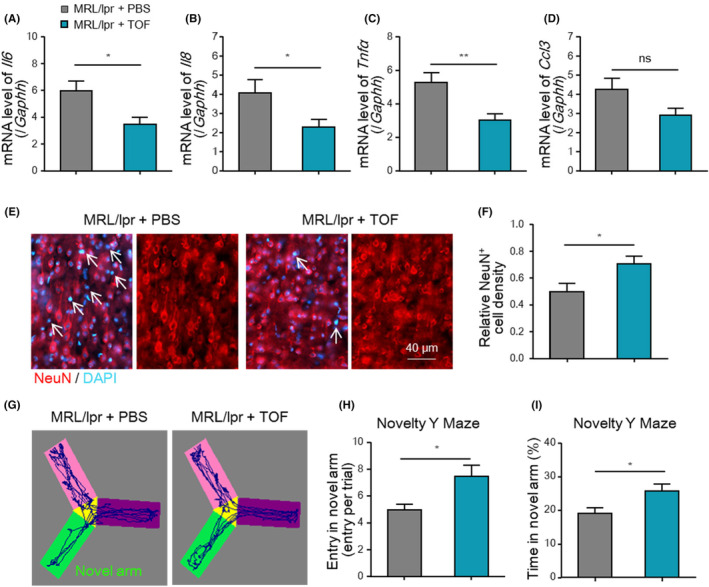
Tofacitinib treatment alleviated inflammatory neurodegeneration and cognitive impairment in MRL/lpr mice. (A–D) mRNA levels of *Il6*, *Il8*, *Tnfα* and *Ccl3* in the cortices of tofacitinib‐treated MRL/lpr mice and vehicle‐treated controls (*n* = 5). (E, F) Representative images and quantification of NeuN^+^ neurons in the cortices of the indicated mice (*n* = 5). Scale bar, 40 μm. (G–I) Number of entries and time spent with the head in the Novel arm in the NYM test (*n* = 6). Total time in the NYM: 3 min. The data are presented as the mean ± SEM. *p < 0.05; **p < 0.01 and *t*‐test. NYM, novelty Y maze; ns, not significant; TOF, tofacitinib.

Studies have demonstrated that JAK inhibition can ameliorate kidney injury and disease activity in lupus mice.^22^, ^42^ Thus, we investigated whether administration of tofacitinib, an FDA‐approved JAK inhibitor, exerts neuroprotective effects in lupus mice. As shown in Figure 7A–D, the levels of M1‐associated proinflammatory cytokines were significantly decreased in tofacitinib‐treated mice compared with vehicle‐treated mice. The onset of neuronal apoptosis was delayed in the tofacitinib‐treated mice. Additionally, NeuN^+^ neuronal loss was significantly alleviated in tofacitinib‐treated mice at 20 weeks of age (Figure 7E,F). Functionally, compared with vehicle treatment, tofacitinib treatment enhanced cognitive performance in the EPM test (Figure 7G–I).

Therefore, we speculated that the FDA‐approved drugs minocycline and tofacitinib can ameliorate CNS injuries in lupus by repressing microglial phagocytosis and JAK‐induced M1 polarization.

## 4 DISCUSSION

**FIGURE 8:**
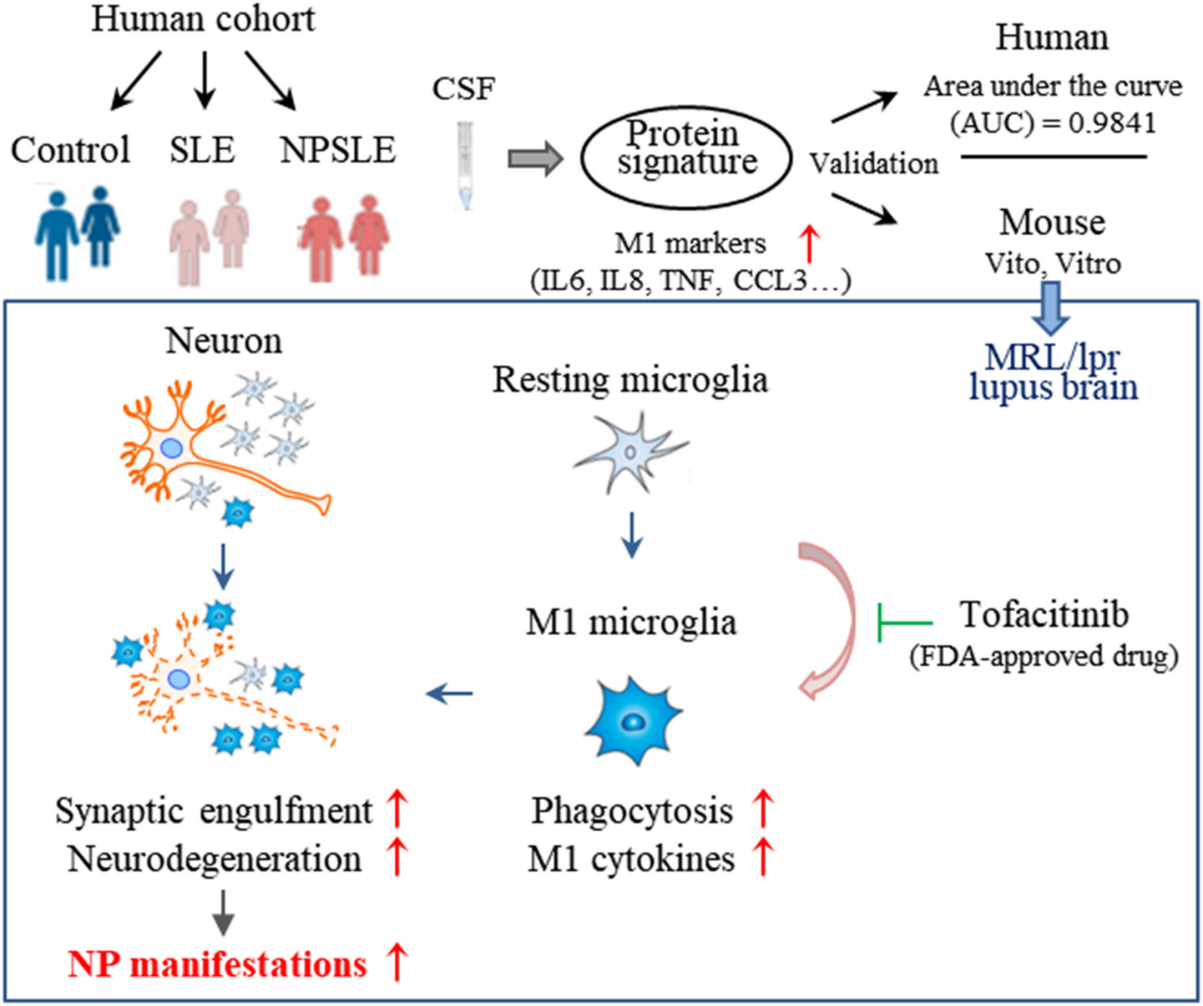
Proposed model of M1 microglia phenotype involved in regulation of synaptic and neuronal degeneration in both neuropsychiatric lupus (NPSLE) patients and MRL/lpr lupus‐prone mice. FDA‐approved drug tofacitinib compromises sdetrimental effects of M1 microglia and accordingly suppresses the neurotoxicity process in lupus brain, providing potential therapeutic strategy in the treatment of NPSLE.

The most important finding of the present study was that classical activation of microglia plays a crucial role in the pathogenesis of NPSLE. Increased M1‐associated proinflammatory cytokine levels and microglial overactivation were observed in both NPSLE patients and NPSLE mouse models. The study also provided a therapeutic strategy to treat NPSLE by targeting microglia. Minocycline and tofacitinib can ameliorate CNS injuries in lupus at different stages, at least partially by repressing microglial phagocytosis and M1 polarization‐mediated inflammatory neuronal damage (Figure 8).

Although lupus is a multifactorial disease, brain involvement occurs early in its course.^2^, ^4^ Therefore, identifying the key pathological mediators in the brain is of great importance for elucidating the aetiology of lupus and developing targeted therapies. Pathological and functional studies of brain samples from patients with SLE have been hindered by both ethical and technical limitations because brain tissue samples are rarely obtained from SLE patients. Moreover, proteomic analysis of CSF samples is limited by the poor sensitivity of available techniques. Our preliminary experiments indicated that at least 10 mL CSF is required for the detection of proteins that are expressed at relatively low levels in the brain but may have high diagnostic potential. Especially for autoimmune diseases, more accurate diagnosis can only be achieved by combining multiple biomarkers. The ability to collect a large amount of CSF from each SLE patient is limited by ethical issues, and it is technically difficult to achieve sensitive, high‐throughput multiple detection. To overcome these issues, we utilized the PEA^27^ to identify the inflammatory protein signature in the CSF of SLE patients, especially those who developed neuropsychiatric symptoms.

We found that the levels of 13 of 54 specific cytokines, including IL6, IL8, TNF, CCL3, MCP2 and OSM and other inflammatory factors, were increased in the CSF of NPSLE patients compared with that of SLE patients without neuropsychiatric symptoms. We further identified a prediagnostic protein signature consisting of IL8, OSM, CCL4, CCL3 and CCL28. Functionally, the upregulated cytokines in the CFS of NPSLE patients were related to M1‐associated proinflammatory effects. This protein signature was further verified in the brains of MRL/lpr mice that developed neuropsychiatric behavioural changes, confirming its biological validity.

Microglia, which are brain‐resident macrophages, produce large amounts of cytokines in the brain and constantly survey the neural environment.^14^, ^43^ When microglia are abnormally activated, their phagocytic activity and secretion of proinflammatory factors are significantly enhanced.^13^ Our previous study showed that microglial overactivation is involved in neurogenesis^44^ and neuroinflammation.^28^, ^31^ In the present study, we found that classically activated microglia were sufficient for neuropsychiatric dysfunction in lupus. Behavioural analysis indicated that MRL/lpr mice exhibited increased anxiety and cognitive defects in the early and active stages of the disease. Microglia activation in both the cortex and hippocampus of the MRL/lpr mouse brain was confirmed by an increase in microglial density, alterations in phagocytic morphology and upregulated expression of proinflammatory cytokines. Inhibition of microglial activity by minocycline significantly alleviated the observed behavioural abnormalities in MRL/lpr mice. These results indicate that microglial activation is essential for the development of NPSLE, as recently reported.^45^, ^46^

To elucidate the molecular mechanisms by which microglia mediate neuropsychiatric dysfunction in lupus, we sorted microglia from MRL/lpr mice and performed RNA sequencing analysis. Our study revealed that microglia in the brains of lupus mice showed specific activation of ‘phagocytosis, recognition’, ‘response to interferon gamma’ and ‘cellular response to lipopolysaccharide’. Indeed, in addition to playing inflammatory roles, microglia were recently revealed to have important regulatory roles in synaptic integrity^47^, ^48^ and refinement.^49^ Increased synaptic pruning/engulfment by microglia contributes to synapse‐related pathology in many CNS disorders.^26^, ^39^, ^50^ In this study, we found that compared with control mice, MRL/lpr mice showed decreased dendritic spine density and increased expression of synaptic markers, such as PSD95, within microglia. Furthermore, treatment with minocycline suppressed microglia‐mediated synaptic engulfment and reduced synaptic loss, eventually alleviating anxiety‐like behaviour in lupus mice. These findings suggest that microglial phagocytosis‐mediated synaptic loss accounts for the early occurrence of anxiety‐like behaviour in lupus mice.

In addition to playing a phagocytic role, microglia can directly induce neuronal death by secreting inflammatory cytokines to aggravate neurodegeneration.^13^, ^51^ An increase in the number of TUNEL‐positive apoptotic neurons was observed in the brains of lupus mice during the active stage.^29^ Accordingly, in this study, we found a significant decrease in the number of NeuN‐positive neurons in the cortices of MRL/lpr mice at 16 weeks of age, accompanied by cognitive impairment. In vitro, neurons cocultured with LPS + IFNγ‐stimulated microglia showed significantly decreased viability and increased apoptosis, even in the absence of direct contact with microglia. Furthermore, treatment with the JAK inhibitor tofacitinib almost abolished the exacerbation of neurotoxicity induced by M1 microglia. More importantly, the suppression of JAK by tofacitinib alleviated microglial activation, neuronal loss and cognitive defects in MRL/lpr mice. This founding is consistent with the reports that blocking the IFNγ‐induced JAK/STAT pathway can revise synaptic loss in viral encephalitis.^26^ Although various JAK inhibitors are being studied, tofacitinib is the first orally available JAK inhibitors to be approved in treating rheumatoid arthritis and other autoimmune diseases.^52^ Mechanically, tofacitinib could inhibit the proliferation of CD4^+^ T cells^53^ and impair the survival rate of CD69^+^CD103^+^ renal‐resident memory T cells in lupus mouse.^54^ These results suggest that the secretion of M1‐associated proinflammatory factors by microglia induces neuronal death and is involved in the later development of cognitive deficits in lupus mice.

Microglial phagocytosis and M1 polarization, as crucial events in synaptic and neuronal damage in lupus, are potential therapeutic targets. Another important finding of this study is that currently available drugs, such as minocycline and JAK inhibitors, exert neuroprotective effects in NPSLE by targeting microglial overactivation. Moreover, both minocycline and tofacitinib can pass through the blood–brain barrier and have been approved by the United States FDA for other purposes, making them promising candidates for pharmacological intervention for NPSLE.

Infiltration of peripheral inflammatory cells into the brain parenchyma or choroid plexus (CP) have been suggested as participants in NPSLE.^55^ We have previous revealed that MRL/lpr strain showed no cellular infiltration in the brain at 6–8 weeks old,^29^ indicating CNS‐resident cells may accounted for the anti‐anxiety effect of minocycline. However, at a later stage of the disease, when a breach in BBB integrity occurs, T and B cells can enter the brain, and also aggravate neuronal death. In complementary experiments, we did find that tofacitinib treatment reduced the number of T cells but not B cells in the CP of MRL/lpr mice compared with vehicle (Figure S1). Thus, we speculated that tofacitinib exerts neuroprotective effect also by reducing the brain infiltration of peripheral cells, rather than a direct role in controlling microglia.

In summary, our study demonstrates that microglial overactivation contributes to NPSLE by inducing synaptic stripping and inflammatory neuronal death. Our findings also provide evidence for the potential therapeutic application of minocycline and tofacitinib in the treatment of NPSLE.

## AUTHOR CONTRIBUTIONS

**Yishan Zhou:** Data curation (equal); methodology (equal); software (equal). **Liang Chen:** Conceptualization (equal); methodology (equal); resources (equal); software (equal); writing – review and editing (equal). **Xiulan Zheng:** Data curation (supporting); formal analysis (equal); methodology (equal); project administration (supporting); validation (supporting). **Qijun Fang:** Data curation (equal); writing – review and editing (equal). **Yunzhi Qian:** Data curation (equal); methodology (supporting); software (equal). **Tianshu Xu:** Investigation (supporting); resources (equal); supervision (supporting); writing – review and editing (supporting). **Jun Liang:** Formal analysis (supporting); investigation (supporting); resources (equal); visualization (supporting). **Huajun Zhang:** Writing – review and editing (equal). **Xiaojuan Han:** Conceptualization (lead); formal analysis (lead); funding acquisition (lead); project administration (lead); writing – original draft (lead). **Lingyun Sun:** Conceptualization (lead); funding acquisition (lead); investigation (lead); supervision (equal); writing – review and editing (lead).

## FUNDING INFORMATION

This research was supported by grants from the National Key R&D Program of China (2020YFA0710800), the Key Program of National Natural Science Foundation of China (81930043, 82330055), the National Natural Science Foundation of China (82273907) and the Fund for Distinguished Young Scientists in Nanjing (JQX21003), Nanjing Youth Training Program of Traditional Chinese Medicine (ZYQ20066), Natural Science Foundation Project of Nanjing University of Traditional Chinese Medicine (28518).

## CONFLICT OF INTEREST STATEMENT

The authors declare no conflicts of interest.

## Supporting information


Figure S1.

Table S1.

Table S2.

Table S3.

Table S4.

## Data Availability

Data Availability StatementThe RNA sequencing data supporting the conclusions of this article have been deposited into the NCBI GEO data repository under accession number GSE201282 (https://www.ncbi.nlm.nih.gov/geo/info/linking.html). Other data that support the findings of this study are available from the corresponding author.
