# Supplementary material for: Microglia orchestrate synaptic and neuronal stripping: Implication in neuropsychiatric lupus
Source: J Cell Mol Med. 2024 Mar 17;28(7):e18190. doi: 10.1111/jcmm.18190 (PMC10945089; doi:10.1111/jcmm.18190)
Supplement: Supplementary file 1 — Figure S1. [file JCMM-28-e18190-s003.docx]

Supplementary Materials for

**Microglia** **orchestrate synaptic and neuronal stripping: Implication in neuropsychiatric lupus**

Yishan Zhou^1,2#^, Liang Chen^3#^, Xiulan Zheng^4#^, Qijun Fang^5#^, Yunzhi Qian^6^, Tianshu Xu^5^, Jun Liang^2^, Huajun Zhang^5^, Xiaojuan Han^1,2,5*^and Lingyun Sun^1,2,4*^

^1^ Department of Traditional Chinese Medicine, Department of Rheumatology and Immunology, Nanjing Drum Tower Hospital, the Affiliated Hospital of Nanjing University Medical School, Nanjing, China; Nanjing Drum Tower Hospital Clinical College of Nanjing University of Chinese Medicine;

^2^ Department of Rheumatology and Immunology, Nanjing Drum Tower Hospital Clinical College of Nanjing Medical University, Nanjing, China;

^3^ Department of Gynecology, The First Affiliated Hospital of Nanjing Medical University, Nanjing, China;

^4^ School of Pharmacy, Macau University of Science and Technology, Macau, China;

^5^ Department of Traditional Chinese Medicine, Nanjing Drum Tower Hospital, the Affiliated Hospital of Nanjing University Medical School, Nanjing, China;

^6^ Department of Nutrition, Gillings School of Global Public Health, University of North Carolina at Chapel Hill, North Carolina, USA.

^#^ Y.Z., L.C., X.Z., and Q.F. contributed equally to this work

^*^ Correspondence: [hxj-719@163.com](mailto:hxj-719@163.com) (X.H.) and lingyunsun@nju.edu.cn (L.S.)

**This file includes:**

Supplementary Figure 1

Supplementary Table legends

**Supplementary Figure 1**

**
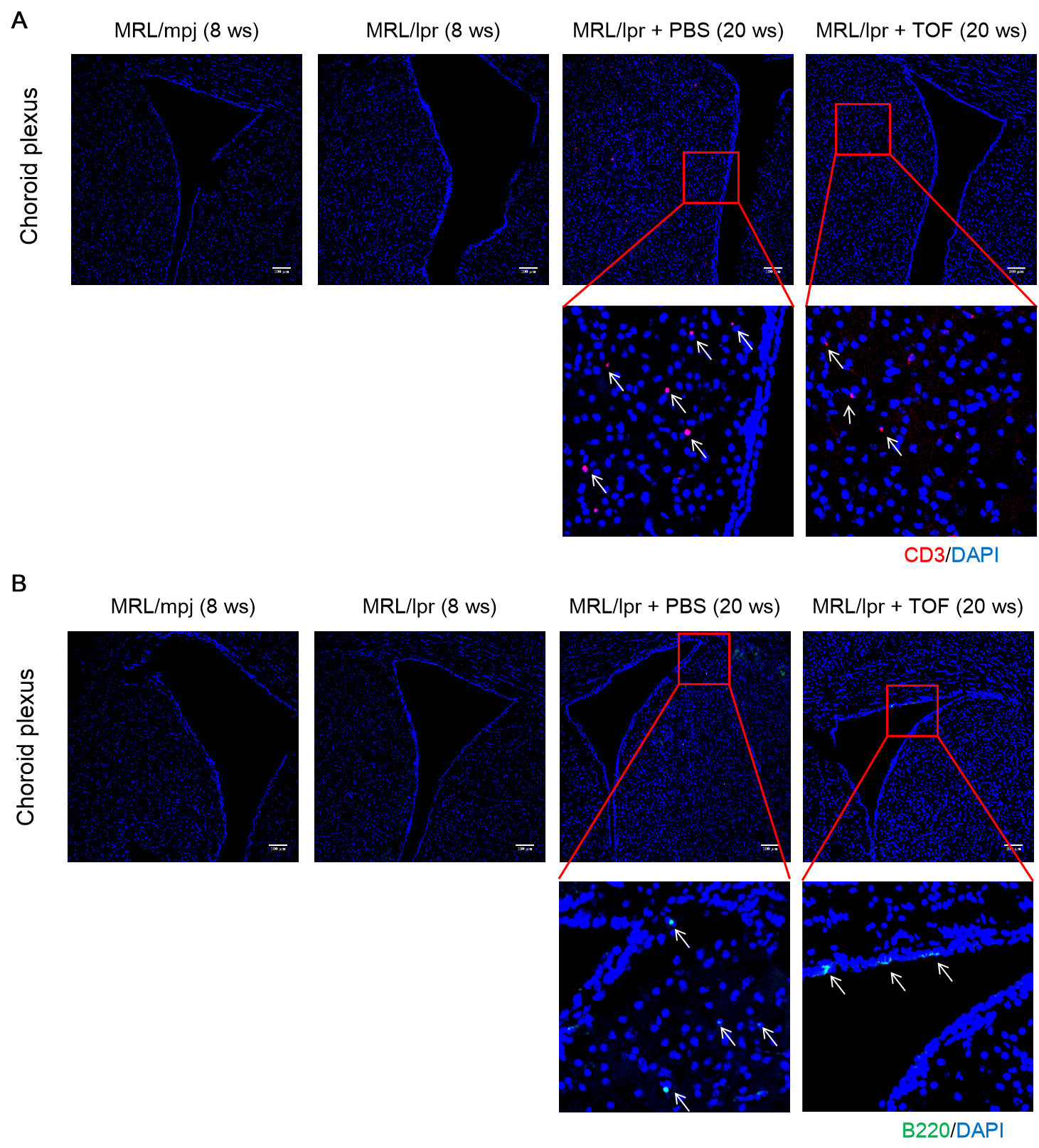
Supplementary Figure 1** Tofacitinib treatment reduced the infiltration of T cells but not B cells into the CP of MRL/lpr mice.

**(A-B)** Representative images of immunofluorescent staining for CD3 **(A)** and B220 **(B)** in the choroid plexus of the indicated mice. Scale bar, 100 μm. TOF, tofacitinib.

**Supplementary Table legends**

**Supplementary Table 1**. Patient data for CSF samples used in this study.

**Supplementary Table 2**. Inflammatory cytokines in CSF from SLE versus control individuals detected by Olink Proteomics.

**Supplementary Table 3.** Biological pathways enriched in microglia sorted from MRL/lpr versus MRL/mpj mice by GSEA analysis with KEGG modules.

**Supplementary Table 4.** Differential expressed genes enriched in JAK/STAT signaling pathway with false discovery rate (FDR) q-value <0.05 in microglia sorted from MRL/lpr versus MRL/mpj mice.
